# Distribution pattern and prognosis of metastatic lymph nodes in cervical posterior to level V in nasopharyngeal carcinoma patients

**DOI:** 10.1186/s12885-020-07146-z

**Published:** 2020-07-17

**Authors:** Chaoyang Jiang, Hui Gao, Ling Zhang, Hua Li, Tao Zhang, Ji Ma, Bisheng Liu

**Affiliations:** 1 Department of Radiation Oncology, The General Hospital of Western Theater Command, Chengdu, Sichuan Province 610083 PR China; 2 Department of Medical Oncology, West China Hospital, Sichuan University, Chengdu, Sichuan Province 610041 PR China; 3 Department of Radiation Oncology, Sichuan Cancer Hospital and Research Institute, University of Electronic Science and Technology of China, Chengdu, Sichuan Province 610041 PR China

**Keywords:** Nasopharyngeal carcinoma, Lymph node metastasis, Posterior to level V, Radiotherapy, Prognosis

## Abstract

**Background:**

Lymph node metastasis in the cervical region posterior to level V (PLV) can occurs in patients with nasopharyngeal carcinoma (NPC), but the significance of lymph node metastasis in this region and the delineation of the radiotherapy target area have not been reported. We aimed to explore the distribution pattern and prognosis of metastatic lymph nodes in the PLV region in patients with NPC.

**Methods:**

We retrospectively studied 605 cases of NPC diagnosed by pathological detection from December 2011 to November 2017. The nodal distribution at each level was assessed in accordance with the Radiation Therapy Oncology Group (RTOG) guidelines proposed in 2013. The central points of the metastatic lymph nodes of the PLV region in the patients were recreated proportionally on the CT images of a standard patient with N0 NPC in reference to the normal anatomy of the PLV area. The correlation between the PLV region and the other levels, the nodal location, and the characteristics and prognosis of the PLV region were analyzed.

**Results:**

Lymph node metastasis occurred in 557 (92.06%) of 605 patients. There were 30 patients (4.95%) with lymph node metastasis in the PLV region. A total of 49 metastatic lymph nodes from the PLV region were counted, and the mean vertical distance of the central point of each lymph node from the anterior surface of the trapezius muscle was 14 mm. Linear regression correlation analysis suggested that lymph node metastasis in the PLV region was associated with ipsilateral level IVa (*P* = 0.018), level Va, level Vb, and level Vc lymph node metastasis (all *P* <  0.001). The 5-year OS, PFS, LRFS, and DMFS of 29 patients with lymph node metastasis in the PLV region were 41.6, 27.7, 89.1, and 47.3%, respectively. Multivariate analysis showed that lymph node metastasis in the PLV region was an independent prognostic factor for DMFS (*P* <  0.05).

**Conclusion:**

NPC patients with lymph node metastasis in the PLV region had a poor prognosis and a high risk of distant metastasis. We recommend that the margin of the PLV region may be a new cervical lymph node segment for NPC.

## Background

Nasopharyngeal carcinoma (NPC) is a malignant tumor of the head and neck. Approximately 80% of NPCs occur in Southeast Asia and South China, including Guangdong, Guangxi, and Hunan Provinces [1]. Since the early symptoms of NPC are not obvious, many patients reach the advanced stage of the disease, and the clinical treatment effect is very poor [2]. Despite improvements in its detection, surgical resection, and radiotherapy, the mortality of NPC is still very high. Currently, radiotherapy is the main treatment for NPC. Radiotherapy combined with chemotherapy or surgery can effectively improve the survival rate of patients with NPC [3]. Importantly, accurate delineation of the radiotherapy target area is key to delivering precise treatment and reducing the side effects for patients with NPC.

**Fig. 1:**
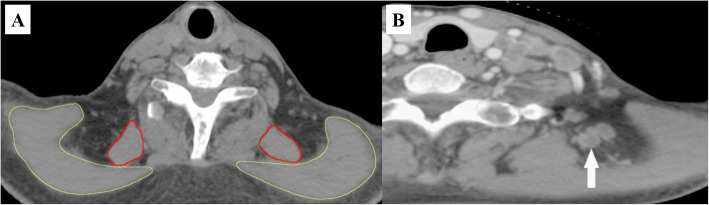
**a**. The PLV region in the CT scan. The PLV region is between the trapezius muscle and the scapular levator. The yellow line presents the trapezius, and the red line presents the levator scapulae. **b**. Metastatic lymph nodes in the PLV region. A typical picture of the metastatic lymph nodes in the PLV region is shown. The white arrows indicate the metastatic lymph nodes

The lymph node metastasis rate of NPC is approximately 80%, which not only affects the clinical stage and radiotherapy plan of NPC but is also one of the main influencing factors of prognosis [4, 5]. In 2013, the new European version of the “National Head and Neck Cancer Cervical Lymph Node Division Guide” (referred to as the 2013 Guideline) not only elaborated the boundaries of each subarea but also further standardized the delineation of radiotherapy target areas of head and neck tumors [6]. In the 2013 Guideline, level V nodes were refined into levels Va, Vb, and Vc, where the anterior border of the trapezius and 1 cm anterior to the serratus anterior muscles were defined as the posterior border of levels Va, Vb and Vc [6]. However, the 2013 Guideline did not describe the cervical region posterior to level V (PLV) (the region between the trapezius muscle and the scapular levator). There are some patients with lymph node metastasis in the PLV region (Fig. 1). Existing studies do not suggest a reference for the delineation of the radiotherapy target area of the PLV region for NPC patients, and the prognosis of NPC patients with lymph node metastasis in the PLV region is unclear [7–10].

In this study, we retrospectively studied the clinical data of 605 patients with NPC, analyzed the extent of cervical lymph node metastasis, and explored the distribution pattern and prognosis of metastatic lymph nodes in the PLV region. Our study provides a useful reference for the delineation of the radiotherapy target area of the PLV region as well as a further revision of the head and neck lymph node division and N stage.

## Methods

### Patient population

This study was approved by the Ethics Committee of The General Hospital of Western Theater Command and Sichuan Cancer Hospital and Institute. We retrospectively reviewed the records of 605 patients with NPC from December 2011 to November 2017 in two hospitals. All patients had been pathologically confirmed as having NPC. Tumor stages and disease grades were classified according to the 2017 edition for the staging of NPC in China [11]. All patients underwent a CT cross-sectional enhanced scan before treatment. The scan range was cranial apex to 2 cm below the sternoclavicular joints, and the thickness of each slice was 3 mm.

### Diagnostic criteria for cervical lymph node metastasis

All patients’ CT and MRI images were reviewed and interpreted by two experienced radiological experts. The criteria for neck lymph node levels are based on the 2013 updated consensus guidelines for neck lymph node levels. The diagnostic criteria for cervical lymph node metastasis were as follows: 1) minimal diameter of lymph nodes on the largest cross-sectional image ≥10 mm [12]; 2) central necrosis or ring enhancement; 3) ≥ 3 lymph nodes in one high-risk area, and a minimum diameter of the largest cross-section of ≥8 mm; 4) lymph node extracapsular invasion, including irregular enhancement of the edge of the lymph node, partial or total disappearance of the peripheral fat space, and lymph node fusion; 5) a minimum diameter of the largest cross-section of the retropharyngeal lymph node of ≥5 mm; 6) a shrunken lymph node after radiotherapy and chemotherapy. One of the above criteria can be judged as an eligibility criterion.

### Delineation of the center point of the lymph node at the PLV

A case of N0 NPC was selected as the standard for a CT simulation scan. According to its anatomical structure and proportion, the central points of the metastatic lymph nodes in the PLV region were outlined on a CT image from the standard case. The central point is defined as the geometric center of the metastatic lymph node. When an individual fused lymph node could not be distinguished, the common geometric center of the observed fused lymph nodes was drawn. The epicenter of every node was contoured by marking the geometric center with a pen tip with a diameter of 5 mm. A horizontal line was drawn on the anterior surface of the trapezius to measure the vertical distance between the center point of each metastatic lymph node in the PLV region and the horizontal line, and the vertical distance of the fusion lymph nodes in the PLV region was measured between the common geometric center of the lymph nodes and the horizontal line.

### Treatment strategy and follow-up

Patients with stage I cancer received radiotherapy alone. Patients with stage II ~ IVa cancer received radiotherapy and chemotherapy with a cisplatin-based regimen. The primary nasopharyngeal tumor and metastatic retropharyngeal lymph nodes were defined as GTVnx. Cervical metastatic lymph nodes were defined as GTVnd. The clinical target volume (CTV1) was defined as a high-risk area that included the GTVnx with a 5–10 mm margin, the whole nasopharynx, GTVnd and the level II and III cervical lymphatic drainage regions. CTV2 was defined as a low-risk area that encompassed CTV1 and the retropharyngeal lymph nodal regions, the base of skull, the anterior half of clivus, the parapharyngeal space, the pterygoid fossa, the inferior sphenoid sinus, the posterior edge of the nasal cavity, the maxillary sinuses and the lower neck. CTV1 and CTV2 volumes underwent a volumetric expansion of 3–5 mm to create PTV1 and PTV2. The prescribed radiation doses of each target volume were as follows: 66–72 Gy for GTVnx, 64–70 Gy for GTVnd, 56–64 Gy for PTV1, and 50–56 Gy for PTV2. All patients received IMRT and irradiated with 1 fraction per day, 5 days per week, for a total of 30–33 fractions. The radiation dose limits for critical structures were within the tolerance recommended by the RTOG 0225 protocol. All patients were followed up by hospitalization, outpatient visits, and telephone enquiries until December 2018. Follow-up examinations included the following: nasopharyngeal and cervical MRI, fiber nasopharyngoscopy, abdominal ultrasound, and chest CT.

### Statistical analysis

All data were analyzed using the SPSS 20.0 software. Linear regression was performed to identify the correlation between the PLV region and the remaining lymph node levels. The Kaplan-Meier method was employed to calculate the survival rate, and the log-rank method was used to compare survival curves between groups. A Cox hazard model with enter method was used to perform multivariate analysis. Overall survival (OS), progression-free survival (PFS), local recurrence-free survival (LRFS), and distant metastasis-free survival (DMFS) were analyzed. *P* values of less than 0.05 were considered statistically significant.

## Results

### Patient characteristics and prognosis

Of the 605 patients with pathologically confirmed NPC, 433 were males and 172 were females. The median age of the patients was 48 years old (12–81 years old), 97.52% of the pathological types were WHO II-III, and 2.47% of the pathological types were WHO type I. The counts and percentages of patients with T1, T2, T3, and T4 NPC were 156 (25.78%), 120 (19.83%), 161 (26.61%), and 168 (27.76%), respectively; and the counts and percentages of patients with N0, N1, N2, and N3 NPC were 48 (7.93%) and 165 (27.27%), 303 (50.08%), 89 (14.71%), respectively. The number and percentages of patients in stages I, II, III, IVa and IVb were 22 (3.63%), 86 (14.21%), 250 (41.32%), 237 (39.17%) and 10 (1.65%), respectively (Table 1). In all patients, 10 patients were in stage IVb, and 4 patients gave up treatment during radiotherapy. A total of 591 patients were followed up for 8–81 months with a median of 37 months, and the 5-year OS, PFS, LRFS, and DMFS were 80.1, 69.4, 88.4, and 83.9%, respectively. Seventy-four patients died, and the main cause of death was distant metastasis, followed by local recurrence and hemorrhage of the nasopharynx. Forty-two cases had local recurrence, mainly in the nasopharyngeal cavity, skull base bone, carotid sheath area, intracranial cavernous sinus area, etc. Seventy-five cases had distant metastasis, most commonly in the liver, lungs and bones. Patients with a single metastasis site were rare, and most patients had two or three sites with simultaneous metastasis.

**Table 1:** Patient characteristics

Characteristic	Number of patients (*n*)
Gender	
Male	433 (71.57%)
Female	172 (28.42%)
Age (years)	
< 45	209 (34.54%)
≥ 45	396 (65.45%)
Histology	
WHO I	15 (2.47)
WHO II- III	590 (97.52)
T stage	
T1	156 (25.78%)
T2	120 (19.83%)
T3	161 (26.61%)
T4	168 (27.76%)
N stage	
N0	48 (7.93%)
N1	165 (27.27%)
N2	303 (50.08%)
N3	89 (14.71%)
TNM stage	
I	22 (3.63%)
II	86 (14.21%)
III	250 (41.32%)
IVa	237 (39.17%)
IVb	10 (1.65%)

### Cervical lymph node metastasis

In the 605 patients, 557 patients (92.06%) had cervical lymph node metastasis (Supplementary Table 1). The top four levels with the highest probability of lymph node metastasis were IIb (77.85%), VIIa (73.05%), IIa (60.0%), and III (41.48%). The levels with less than a 5% probability of the lymph node metastasis was IVb (1.98%), Vc (1.48%), VIIb (0.82%), and VIII (0.49%), and no lymph node metastasis was found in levels Ia, VI, IX and X. There were 12 patients with lymph node metastasis in level IVb, and these patients also had lymph node metastasis in the level IVa. Nine patients had lymph node metastasis in level Vc, and these patients were also accompanied by lymph node metastasis in level Vb. Three patients with lymph node metastasis in level VIII were associated with lymph node metastasis in levels Ib, II, and III, and one patient with lymph node metastasis in level II showed local necrosis and lymph node fusion.

### Distribution characteristics of metastatic lymph nodes in the PLV region

**Fig. 2:**
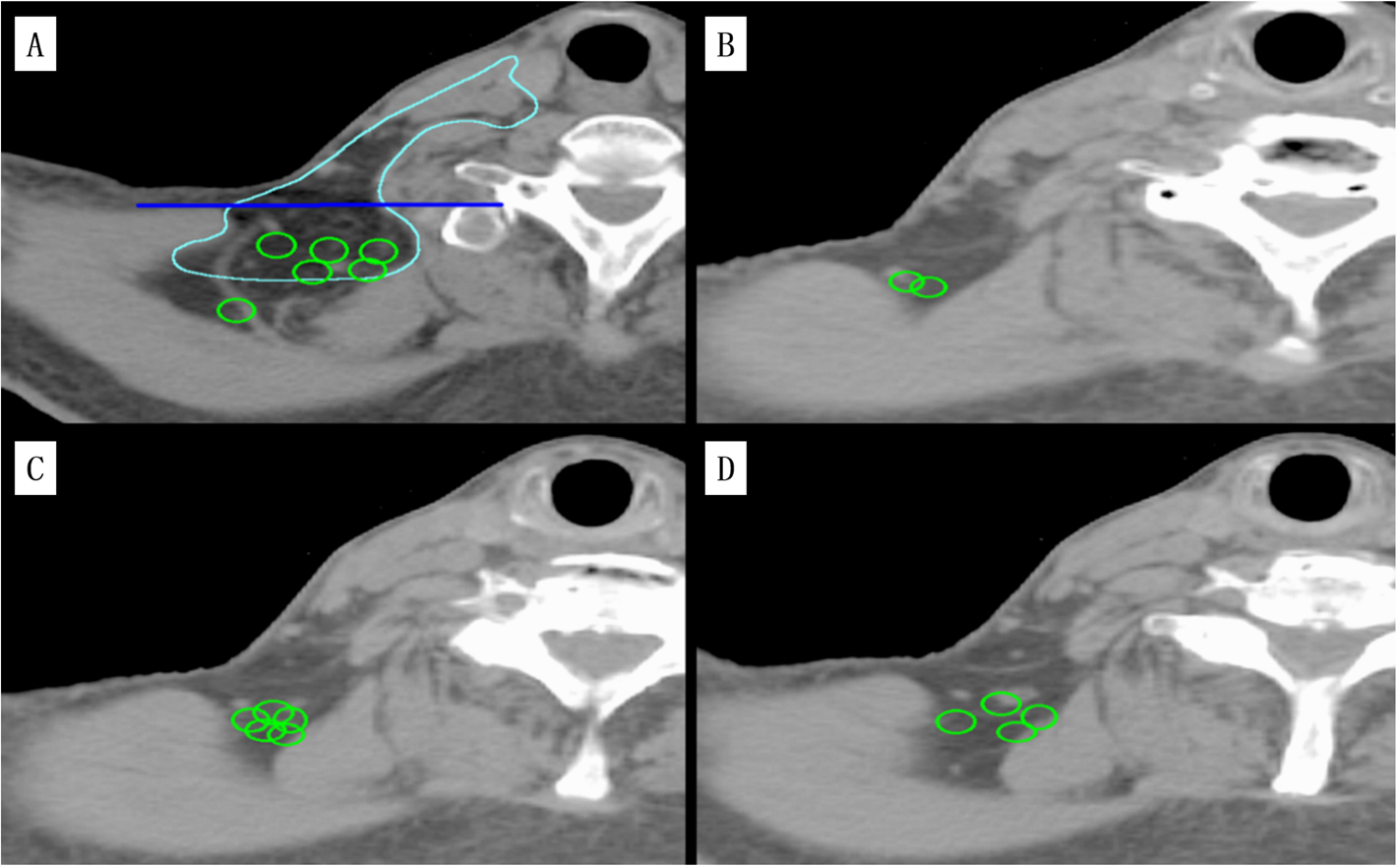
Schematic diagram of the distribution of metastatic lymph nodes in the PLV region. The blue line is the horizontal line through the anterior surface of the trapezius, which was used to calculate the vertical distance between the central points of the metastatic lymph nodes in the PLV region and the horizontal line. The light blue line is the delineation of the PLV region with the ipsilateral metastatic lymph nodes of levels Va, Vb, and Vc. The green circle indicates some metastatic lymph nodes of the PLV region that were recreated proportionally on the CT images of a standard patient to further understand the location of their distribution

In the whole group of 605 patients, 30 patients (4.95%) showed lymph node metastasis in the PLV region (Supplementary Table 2). There was a total of 49 metastatic lymph nodes, including 25 metastatic lymph nodes in the left neck and 24 metastatic lymph nodes in the right neck. In one patient, lymph node metastasis in the PLV region occurred simultaneously on both sides of the neck. There were 23 metastatic lymph nodes with a short diameter of less than 10 mm, 22 metastatic lymph nodes with a short diameter of 11–20 mm, and 4 metastatic lymph nodes with a short diameter of 21–30 mm. The median vertical distance of the center point of each metastatic lymph node from the anterior surface of the trapezius muscle in the standard NPC patient was 14 mm (3–37 mm). There were 25 lymph nodes with a vertical distance of less than 10 mm, 14 lymph nodes with a vertical distance between 11 and 20 mm, 7 lymph nodes with a vertical distance between 21 and 30 mm, and 3 lymph nodes with a vertical distance of more than 31 mm. The centers of 93.87% (46/49) of the metastatic lymph nodes in the PLV region were located less than 25 mm from the anterior surface of the trapezius muscle. The distribution of the metastatic lymph nodes in the PLV region is shown in Supplementary Table 3. The location of the corresponding CT layer of the standard NPC patient is shown in Fig. 2.

### Correlation analysis of lymph node metastasis in the PLV region

To analyze the relationship between lymph node metastasis in the PLV region and other cervical lymph node metastasis, linear regression analysis was used. The lymph node metastasis in the PLV region was used as the dependent variable, and the remaining lymph node regions were included as independent variables in the analysis. The results showed that the lymph node metastasis of the PLV region was associated with the ipsilateral IVa (*P* = 0.018), Va, Vb and Vc levels (all *P* <  0.001), and no correlations were found for the other variables (Table 2).

**Table 2:** The data of linear-regression analysis for neck node

Variable	B	Std.Error	t	*p*	95% CI for B
Ib	0.014	0.036	0.374	0.708	−0.057-0.085
IIa	−0.004	0.016	−0.228	0.820	−0.035-0.028
IIb	− 0.004	0.019	− 0.224	0.823	− 0.042-0.034
III	−0.012	0.018	0.669	0.504	−0.047-0.023
IVa	0.062	0.026	2.366	**0.018**	0.011–0.114
IVb	0.106	0.059	1.776	0.076	−0.011-0.222
Va	0.118	0.021	5.729	**0.000**	0.078–0.159
Vb	0.243	0.038	6.391	**0.000**	0.168–0.318
Vc	0.435	0.071	6.112	**0.000**	0.295–0.574
VIIa	0.017	0.016	1.041	0.298	−0.015-0.048
VIIb	0.001	0.077	0.017	0.987	−0.151-0.153
VIII	−0.162	0.101	−1.597	0.111	−0.360-0.037

Abbreviations: *B* regression coefficient, *t* t test, Dependent Variable: PLV

### Prognosis of patients with lymph node metastasis in the PLV region

The number of patients with lymph node metastasis in PLV region was 30, but 1 patient with stage IVB was excluded and we followed up 29 patients. A total of 29 patients with lymph node metastasis in PLV were followed up for a median of 21 (4 to 60) months. Fourteen patients had distant metastasis, 11 patients died during the follow-up period (death overlapped with distant metastasis), and 2 patients relapsed. The 5-year OS, PFS, LRFS, and DMFS were 41.6, 27.7, 89.1, and 47.3%, respectively.

### Prognosis of patients with N3 NPC with or without lymph node metastasis in the PLV region

**Fig. 3:**
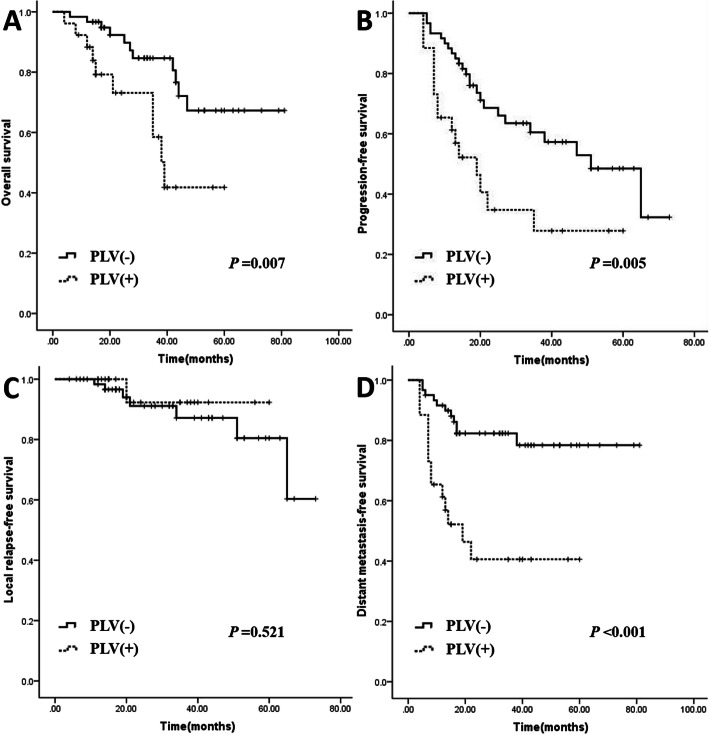
The 5-year survival curve of patients with N3 NPC with or without lymph node metastasis in the PLV region. **a** OS (*P* = 0.007), **b** PFS (*P* = 0.005), **c** LRFS (*P* = 0.521), **d** DMFS (*P* <  0.001)

The number of patients with N3 was 89, but 3 patients with N3 in stage IVB were excluded, so the cases of patients with N3 NPC with or without lymph node metastasis in the PLV region were 26 and 60, respectively. The 5-year OS, PFS, LRFS, and DMFS of the two groups were 41.8 and 67.3% (*P* = 0.007), 27.8 and 48.5% (*P* = 0.005), 92.3 and 80.5% (*P* = 0.521), and 40.6 and 78.4% (*P* <  0.001), respectively (Table 3, Fig. 3).

**Table 3:** The survival data of N3 patients with or without node metastasis of PLV

Variable	5-year OS		5-year PFS		5-year LRFS		5-year DMFS	
	%	*P*	%	*P*	%	*P*	%	*P*
PLV (+) (*n* = 26)	41.8	0.007	27.8	0.005	92.3	0.521	40.6	< 0.001
PLV (−) (*n* = 60)	67.3		48.5		80.5		78.4	

Abbreviations: *PLV (+)* node metastasis with posterior to level V, *PLV (−)* node metastasis without posterior to level V

### Univariate and multivariate analysis

The univariate results showed gender and age were prognostic factors for 5-year OS (all *P* <  0.05), T-stage was a prognostic factor for 5-year LRFS(*P* = 0.003), The N-stage and TNM stage were prognostic factors for 5-year OS, PFS, LRFS, and DMFS (all *P* <  0.05), Involvement of lower neck was a prognostic factor for 5-year OS, PFS, and DMFS (all *P* <  0.001) (Table 4). Involvement of lower neck was refined into levels IVa, IVb, Vb, and Vc and the PLV region in multivariate analysis, and the parameters were designed as two categorical variables (Table 5). Analysis showed that lymph node metastasis in the PLV region was an independent prognostic factor for DMFS (*P* = 0.044) rather than for OS and PFS (Table 5, Supplementary Table 4 and 5).

**Table 4:** Univariate analysis of prognosis in 591 NPC patients

Variable	N	5-year OS (%)	*p*	5-year PFS (%)	*p*	5-year LRFS (%)	*p*	5-year DMFS (%)	*p*
Gender									
Male	423	78.0	0.04	67.5	0.194	89.0	0.619	82.2	0.328
Female	168	85.6		74.1		87.2		87.8	
Age (years)									
< 45	205	86.3	0.039	72.0	0.463	87.3	0.744	84.9	0.846
≥ 45	386	76.8		67.9		89.0		83.2	
T stage									
T_1_ + T_2_	271	82.4	0.28	72.8	0.096	93.1	0.003	85.2	0.802
T_3_ + T_4_	320	77.2		62.4		82.9		81.7	
N stage									
N_0_ + N_1_	211	86.6	0.004	79.8	< 0.001	91.7	0.025	90.4	0.002
N_2_ + N_3_	380	73.9		58.8		86.0		78.9	
TNM stage									
I + II	108	89.6	0.006	84.4	< 0.001	94.9	0.014	92.3	0.015
III + IVa	483	76.7		64.6		86.3		81.1	
Involvement of lower neck levels									
Yes	90	57.6	< 0.001	41.7	< 0.001	82.3	0.07	68.7	< 0.001
No	501	83.5		74.2		89.3		86.7

**Table 5:** Multivariate analysis of DMFS in 591 NPC patients

Variable	B	SE	*P*	HR	95%CI
Involvement of lower neck levels (yes vs. no)					
level IVa	0.241	0.358	0.501	1.273	0.631–2.568
level IVb	0.909	0.564	0.107	2.481	0.821–7.500
level Vb	0.660	0.460	0.151	1.934	0.786–4.763
level Vc	0.102	0.650	0.875	1.107	0.310–3.956
PLV	0.925	0.460	0.044	2.522	1.023–6.214
N stage (N0 + 1 vs. N2 + 3)	−0.296	0.360	0.410	0.744	0.367–1.506
TNM stage (I + II vs. III + IVa)	−0.390	0.475	0.411	0.677	0.267–1.716

## Discussion

Nasopharyngeal carcinoma (NPC) is very prone to lymph node metastasis [13]. Some studies have reported that approximately 40% of patients with NPC have a first symptom of cervical lymphadenopathy [14]. Approximately 60 to 90% of patients with newly diagnosed NPC have lymph node metastasis [15]. Therefore, the segmentation and metastatic characteristics of cervical lymph nodes are of great importance for the delineation of target areas of radiotherapy. In the 2013 Guideline, level V of cervical lymph nodes is refined into levels Va, Vb, and Vc [6]. However, the 2013 Guideline did not describe the PLV gap (the gap between the trapezius muscle and the scapular levator). In the PLV region, metastatic lymph nodes can be found. Unfortunately, this PLV region is often overlooked. The delineation of the radiotherapy target area for the PLV region and the prognosis of NPC patients with lymph node metastasis in the PLV region are still unknown.

Previous studies have shown that the rate of lymph node metastasis is not high in the PLV region; the metastasis rates in the studies [16–18] were 1.1, 2.4 and 2.5%, respectively. In this study, we found that in the whole group of 605 patients, there were 30 patients with lymph node metastasis in the PLV region, and the metastasis rate was 4.95%, slightly higher than in the abovementioned studies, which may be related to the relatively low level of awareness of the disease in patients in western China. In this study, we also found 49 metastatic lymph nodes in the PLV region. Most of the central points of these metastatic lymph nodes were located 25 mm from the anterior surface of the trapezius muscle, and metastatic lymph nodes were located in both the trapezius and scapular levator muscles. Further analysis showed that 132 patients had lymph node metastasis in level Va, including 30 patients with lymph node metastasis in the PLV region. At the same time, we also found that all lymph node metastasis in the PLV region were associated with ipsilateral lymph node metastasis in level Va. Correlation analysis suggested that the lymph node metastasis in the PLV region was also associated with lymph node metastasis in levels IVa, Va, Vb and Vc. Therefore, when NPC patients present with lymph node metastasis especially in level Va, Vb, and Vc but not in the PLV region, it is recommended that the target delineation of posterior boundary of the ipsilateral V region be appropriately moved back to 25 mm behind the anterior surface of the trapezius muscle to prevent lymph node metastasis (Fig. 2); when NPC patients present without lymph node metastasis in these areas, the posterior border of the level V region can be outlined to the anterior surface of the trapezius muscle.

A previous study showed that 5-year OS, PFS, LRFS, and DMFS rates in NPC patients were 77.1, 69.6, 89.8, and 74.1%, respectively [19]. Another study showed 5-year OS, PFS, LRFS, and DMFS rates in NPC patients of 83.3, 76.3, 92.7, 85.5%, respectively [20]. This finding is similar to that of our study, which also showed that the 5-year OS, PFS, LRFS, and DMFS of patients with lymph node metastasis in the PLV were 41.6, 27.7, 89.1, and 47.3%, respectively. Moreover, patients with N3 NPC accompanied by lymph node metastasis in the PLV region have a worse prognosis. Multivariate analysis showed that lymph node metastasis in the PLV region was an independent prognostic factor for DMFS. This further indicates that the prognosis of NPC patients with PLV-region lymph node metastasis is poor, and lymph node metastasis in this region indicates a high risk of distant metastasis. For patients with PLV metastasis, on the one hand, the delineation scope of the radiotherapy target should be expanded, on the other hand, because of the increased risk of distant metastasis (Table 3), it is necessary to strengthen the systemic treatment.

This study has the following limitations: first, a pathological diagnosis of metastatic lymph nodes is lacking, especially in the deep fat gap of the PLV region, and performing histopathology in this region is difficult. Second, since the measurement of the vertical distance was performed on a standard patient, the position of the frontal area of the trapezius muscle may be different depending on the weight, age, and body type of different patients. To minimize these variations, we tried to recreate the position of the central point of each lymph node on the CT images of the standard patient. Third, there is a difference in the fat space between the trapezius muscle and the levator scapulae of in different patients. To clearly show the fat gap between the trapezius muscle and the scapular levator muscle, we selected a patient with a wide gap as the standard patient.

## Conclusion

To the best of our knowledge, in this study, we report for the first time the distribution and metastasis of lymph nodes in the PLV region of NPC patients and provide a reference for the delineation of the lymph node area in the PLV region. In addition, we found that the metastasis rate of lymph nodes in the PLV region was 4.95%, which was close to or even exceeded the metastasis rate in the Ib, IVb, Vb, and Vc regions. However, the 2013 Guideline did not fully consider the PLV region, which may be a missing neck segment. Therefore, we propose to use the PLV region as a new cervical lymph node level as follows: cranial boundary: the caudal edge of the cricoid cartilage; caudal boundary, the level of the clavicle or serratus anterior muscle; the anterior boundary: the anterior surface of the trapezius muscle; posterior boundary: the intersection of the trapezius muscle and the scapula levator; medial boundary: the lateral edge of the scapular levator; and lateral boundary: the medial edge of the trapezius muscle.

## Supplementary information

**Additional files 1: Supplementary Table 1.** Patterns of cervical nodal metastasis of nasopharyngeal carcinoma

**Additional files 2: Supplementary Table 2.** Baseline characteristics of patients with metastasis of posterior to level V

**Additional files 3 Supplementary Table 3**. Patterns of cervical nodal metastasis of posterior to level V

**Additional files 4: Supplementary Table 4.** Multivariate analysis for OS in 591 NPC patients

**Additional files 5: Supplementary Table 5.** Multivariate analysis for PFS in 591 NPC patients

## Data Availability

The datasets used and/or analysed during the current study are available from the corresponding author on reasonable request.

## Abbreviations

PLV: Posterior to level V
NPC: Nasopharyngeal carcinoma
RTOG: Radiation Therapy Oncology Group
CTV: Clinical target volume
OS: Overall survival
PFS: Progression-free survival
LRFS: Local recurrence-free survival
DMFS: Distant metastasis-free survival
