# Supplementary material for: Distribution pattern and prognosis of metastatic lymph nodes in cervical posterior to level V in nasopharyngeal carcinoma patients
Source: BMC Cancer. 2020 Jul 17;20:667. doi: 10.1186/s12885-020-07146-z (PMC7366893; doi:10.1186/s12885-020-07146-z)
Supplement: Supplementary file 1 — Additional files 1: Supplementary Table 1. Patterns of cervical nodal metastasis of nasopharyngeal carcinoma [file 12885_2020_7146_MOESM1_ESM.docx]

Supplementary table 1: Patterns of cervical nodal metastasis of nasopharyngeal carcinoma

| Neck node level | Number（%） |
| --- | --- |
| Ⅰa | 0(0.0) |
| Ⅰb | 25(4.13) |
| Ⅱa | 363(60.0) |
| Ⅱb | 471(77.85) |
| Ⅲ | 251(41.48) |
| Ⅳa | 77(12.72) |
| Ⅳb | 12(1.98) |
| Va | 132(21.81) |
| Vb | 35(5.78) |
| Vc | 9(1.48) |
| Ⅵ | 0(0.0) |
| VIIa | 442(73.05) |
| VIIb | 5(0.82) |
| Ⅷ | 3(0.49) |
| Ⅸ | 0(0.0) |
| Ⅹ | 0(0.0) |
