# Supplementary material for: Distribution pattern and prognosis of metastatic lymph nodes in cervical posterior to level V in nasopharyngeal carcinoma patients
Source: BMC Cancer. 2020 Jul 17;20:667. doi: 10.1186/s12885-020-07146-z (PMC7366893; doi:10.1186/s12885-020-07146-z)
Supplement: Supplementary file 2 — Additional files 2: Supplementary Table 2. Baseline characteristics of patients with metastasis of posterior to level V [file 12885_2020_7146_MOESM2_ESM.docx]

Supplementary table 2: Baseline characteristics of patients with metastasis of posterior to level V

| Gender | Number（%） |
| --- | --- |
| Male | 28(93.33) |
| Female | 2(6.66) |
| T stage |  |
| T1 | 7(23.33) |
| T2 | 7(23.33) |
| T3 | 8(26.66) |
| T4 | 8(26.66) |
| N stage |  |
| N2 | 3(10.00) |
| N3 | 27(90.00) |
| TNM stage |  |
| Ⅲ | 3(10.00) |
| Ⅳa | 26(86.66) |
| Ⅳb | 1(3.33) |
