# Supplementary material for: Distribution pattern and prognosis of metastatic lymph nodes in cervical posterior to level V in nasopharyngeal carcinoma patients
Source: BMC Cancer. 2020 Jul 17;20:667. doi: 10.1186/s12885-020-07146-z (PMC7366893; doi:10.1186/s12885-020-07146-z)
Supplement: Supplementary file 3 — Additional files 3 Supplementary Table 3. Patterns of cervical nodal metastasis of posterior to level V [file 12885_2020_7146_MOESM3_ESM.docx]

Supplementary table 3: Patterns of cervical nodal metastasis of posterior to level V

| Short diameter of lymph nodes (mm) | Left (%) | Right (%) |
| --- | --- | --- |
| ≤10 | 12(24.48) | 11(22.44) |
| 11-20 | 9(18.36) | 13(26.53) |
| 21-30 | 4(8.16) | 0(0) |
| The vertical distance from the anterior border of trapezius (mm)  ≤10 | Left (%)  11(22.44) | Right (%)  14(28.57) |
| 11-20 | 8(16.32) | 6(12.24) |
| 21-30 | 4(8.16) | 3(6.12) |
| ≥31 | 2(4.08) | 1(2.04) |
| sum | 25(51.02) | 24(48.97) |
