# Supplementary material for: Distribution pattern and prognosis of metastatic lymph nodes in cervical posterior to level V in nasopharyngeal carcinoma patients
Source: BMC Cancer. 2020 Jul 17;20:667. doi: 10.1186/s12885-020-07146-z (PMC7366893; doi:10.1186/s12885-020-07146-z)
Supplement: Supplementary file 4 — Additional files 4: Supplementary Table 4. Multivariate analysis for OS in 591 NPC patients [file 12885_2020_7146_MOESM4_ESM.docx]

Supplementary table 4: Multivariate analysis for OS in 591 NPC patients

| Variable | B | SE | *P* | HR | 95%CI |
| --- | --- | --- | --- | --- | --- |
| Involvement of lower neck levels (yes vs. no)  level IVa  level IVb  level Vb  level Vc  PLV  Gender (male vs. female)  Age (＜45 vs. ≥45 )  N stage (N0+1 vs. N2+3)  TNM stage (Ⅰ+Ⅱ vs. Ⅲ+Ⅳa) | 0.665  0.198  0.082  1.208  0.520  -0.566  0.546  -0.246  -0.497 | 0.358  0.626  0.539  0.742  0.529  0.307  0.277  0.334  0.437 | 0.063  0.752  0.880  0.103  0.325  0.065  0.049  0.462  0.255 | 1.945  1.219  1.085  3.348  1.683  0.568  1.726  0.782  0.609 | 0.964-3.927  0.357-4.155  0.377-3.123  0.782-14.334  0.596-4.748  0.311-1.037  1.004-2.968  0.407-1.505  0.258-1.433 |
