# Supplementary material for: Distribution pattern and prognosis of metastatic lymph nodes in cervical posterior to level V in nasopharyngeal carcinoma patients
Source: BMC Cancer. 2020 Jul 17;20:667. doi: 10.1186/s12885-020-07146-z (PMC7366893; doi:10.1186/s12885-020-07146-z)
Supplement: Supplementary file 5 — Additional files 5: Supplementary Table 5. Multivariate analysis for PFS in 591 NPC patients [file 12885_2020_7146_MOESM5_ESM.docx]

Supplementary table 5: Multivariate analysis for PFS in 591 NPC patients

| Variable | B | SE | *P* | HR | 95%CI |
| --- | --- | --- | --- | --- | --- |
| Involvement of lower neck levels (yes vs. no)  level IVa  level IVb  level Vb  level Vc  PLV  N stage (N0+1 vs. N2+3)  TNM stage (Ⅰ+Ⅱ vs. Ⅲ+Ⅳa) | 0.531  0.545  0.222  0.632  0.667  -0.355  -0.530 | 0.250  0.455  0.379  0.549  0.377  0.256  0.342 | 0.034  0.231  0.558  0.250  0.077  0.166  0.121 | 1.701  1.724  1.248  1.881  1.948  0.701  0.588 | 1.042-2.778  0.707-4.205  0.594-2.622  0.641-5.519  0.931-4.077  0.425-1.158  0.301-1.150 |
